# CHK2 is essential for spindle assembly and DNA repair during the first cleavage of mouse embryos

**DOI:** 10.18632/aging.103267

**Published:** 2020-06-02

**Authors:** Xiao-Han Li, Wen-Jing Li, Jia-Qian Ju, Meng-Hao Pan, Yao Xu, Ming-Hong Sun, Mo Li, Shao-Chen Sun

**Affiliations:** 1 College of Animal Science and Technology, Nanjing Agricultural University, Nanjing 210095, China; 2 Center for Reproductive Medicine, Peking University Third Hospital, Beijing 100191, China

**Keywords:** CHK2, early embryo, spindle, chromosome, DNA damage

## Abstract

The quality of the early embryo is critical for embryonic development and implantation. Errors during cleavage lead to aneuploidy in embryos. As a cell cycle checkpoint protein, CHK2 participates in DNA replication, cell cycle arrest and spindle assembly. However, the functions of CHK2 in early development of the mouse embryo remain largely unknown. In this study, we show that CHK2 is localized on the spindle in metaphase and mainly accumulates at spindle poles in anaphase/telophase during the first cleavage of the mouse embryo. CHK2 inhibition led to cleavage failure in early embryonic development, accompanied by abnormal spindle assembly and misaligned chromosomes. Moreover, the loss of CHK2 activity increased the level of cellular DNA damage, which resulted in oxidative stress. Then, apoptosis and autophagy were found to be active in these embryos. In summary, our results suggest that CHK2 is an essential regulator of spindle assembly and DNA repair during early embryonic development in mice.

## INTRODUCTION

Development of the early embryo in mammals is initiated by the fertilization of an oocyte by sperm [1]. Following fertilization, the mammalian oocyte completes second meiosis to form a zygote. The zygote undergoes the first cleavage to generate two cells under maternal regulation [2]. Subsequently, the embryonic genome is activated, and the embryo undergoes continuous cleavage to develop into a 4-cell embryo, 8-cell embryo, morula and blastocyst. Then, the embryo differentiates into the trophoblast ectoderm (TE) and inner cell mass (ICM) during the blastocyst stage [3]. Errors during this process lead to embryonic lethality and implantation failure.

The cell cycle is a sequence of events involving the replication of cellular components and the precise separation of daughter cells. In eukaryotes, DNA replication occurs in S phase, and chromosome separation occurs in M phase. The G1 and G2 phases separate the S and M phases, when cells prepare for DNA replication and chromosome separation, respectively [4]. To maintain the integrity of the genome, cells have evolved monitoring programs to ensure DNA replication and accurate chromosome segregation. Cell cycle checkpoints are monitoring mechanisms that supervise the sequence, integrity and fidelity of major events during the cell cycle, which include the control of appropriate cell size, DNA replication, and precise segregation of chromosomes during division [5]. DNA damage can cause cell cycle arrest, which gives cells enough time to undergo repair before moving on to the next stage. The checkpoint mechanism involves a number of highly conserved proteins that sense DNA damage signals [6]. The transcription factor p53 is an important component of the G1-phase checkpoint [7]. CHK1 (checkpoint kinase 1), an effector protein of the G2 DNA damage checkpoint, is involved in regulating DNA damage repair after replication [8, 9]. The checkpoint regulation mechanism in M-phase cells is the spindle assembly checkpoint, which monitors chromosome separation to avoid aneuploidy in daughter cells [10, 11]. BubR1 is a critical spindle assembly checkpoint protein that binds the kinetochore in mitosis or meiosis and regulates accurate chromosome separation [12, 13]. Mad1, another spindle assembly checkpoint regulator, has been reported to inhibit the activity of APC/C, resulting in the arrest of oocytes in the metaphase of meiosis to provide time for both homologous chromosome and sister chromatid alignment [14].

Checkpoint kinase 2 (CHK2), an effector kinase of the intra-S-phase checkpoint, is a mammalian homolog of the *Saccharomyces cerevisiae* Rad53 and *Schizosaccharomyces pombe* Cds1 protein kinases [5, 15]. The CHK2 protein mainly consists of three different functional domains: an SQ/TQ cluster domain (SCD), a forkhead-associated (FHA) domain, and a Ser/Thr kinase domain. The SCD contains five SQ and two TQ motifs, which are targets of the kinase ataxia-telangiectasia mutated (ATM) [16]. The FHA domain plays an important role in DNA damage checkpoint pathways [17]. The Ser/Thr kinase domain contains a Gly-rich region in its N-terminal portion and Asp as a catalytic residue at the active site [18]. CHK2 is involved in a variety of events that include regulation of the DNA replication checkpoint, DNA repair, cell cycle arrest, and autophagy caused by DNA damage [18–21]. In human cells, CHK2 is involved in DNA repair by phosphorylation and regulation of the tumor suppressor breast cancer 1 (BRCA1) [22]. CHK2 also participates in the regulation of p53-dependent apoptosis [23]. In addition to the established role of CHK2 in DNA damage, CHK2 is required for the maintenance of chromosomal stability in mitosis and meiosis [23, 24].

Although CHK2 has been shown to be involved in multiple cellular events in different models, the roles of CHK2 during early embryonic development remain unknown. In the present study, we used a mouse model to show that CHK2 activity is essential for spindle assembly, chromosome alignment, and the control of DNA damage repair during the first cleavage of embryos.

## RESULTS

### CHK2 localization during mouse embryonic development

**Figure 1:**
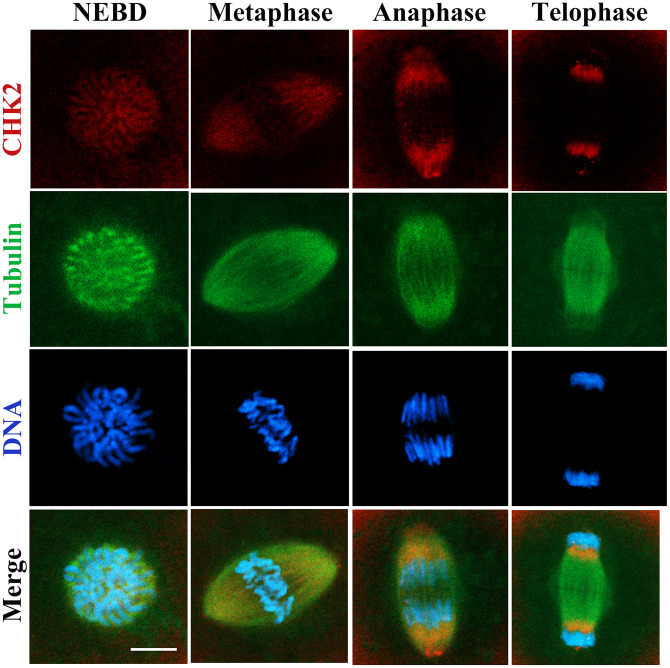
**The localization of CHK2 during early embryonic development in mice.** Embryos at first cleavage were immunolabeled with anti-α-tubulin (green) and anti-CHK2 (red) antibodies, and Hoechst 33342 was used to label DNA (blue). CHK2 was localized near chromosomes after NEBD, and CHK2 accumulated at the spindle area at metaphase, while CHK2 was localized at the spindle poles at anaphase and telophase. Bar = 5 μm.

The subcellular localization of CHK2 at different stages of the first cleavage in mouse embryos was examined by immunofluorescent staining. Our results showed that CHK2 accumulated near chromosomes after nuclear envelope breakdown (NEBD). CHK2 was enriched at the spindle area at metaphase, and when the embryo entered anaphase and telophase, CHK2 was located at the poles of the spindle. No specific CHK2 localization at interphase was observed in the 2-cell embryos; nevertheless, CHK2 accumulated again at the spindle area at metaphase in the 2-cell embryos (Figure 1). This localization pattern indicated a potential relationship between CHK2 and the spindle in mouse embryos.

### Disruption of CHK2 activity inhibited early embryonic development in mice

**Figure 2:**
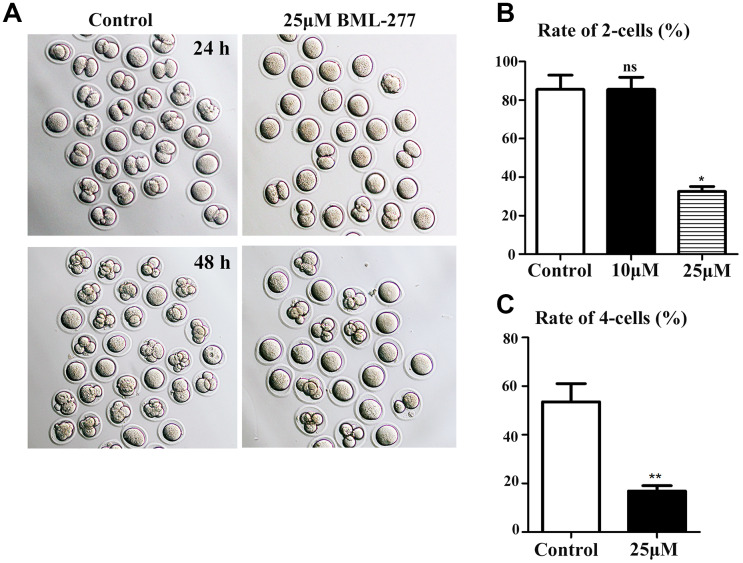
**Disruption of CHK2 activity inhibited cleavage during early embryonic development in mice.** (**A**) Representative image showing the development of early embryos in the 25 μM treatment group and the control group at 24 h and 48 h. (**B**) The rate of 2-cell embryo formation in the 25 μM treatment group was significantly lower than that in the control group (32.5 ± 2.63%, n = 137, 25 μM vs. 85.5 ± 7.42%, n = 158, control, *p* < 0.05). (**C**) The rate of 4-cell embryo formation in the 25 μM treatment group was significantly lower than that in the control group (16.8 ± 2.22%, n = 236, 25 μM vs. 53.6 ± 7.44%, n = 260, control, *p* < 0.01). **significant difference (p < 0.01), *significant difference (p < 0.05).

BML-277 was used to explore the possible roles of CHK2 during early embryonic development in mice. Embryos were treated with BML-277 at different concentrations and cultured for 24 h to analyze the rate of 2-cell embryo formation, and our results showed that most embryos in the 25 μM treatment group failed to undergo the first cleavage. Moreover, most embryos in the 25 μM treatment group failed to develop to the 4-cell stage after 48 h of culture (Figure 2A). The rate of 2-cell embryo formation in the 25 μM treatment group was significantly lower than that in the control group (32.5 ± 2.63%, n = 137, 25 μM vs. 85.5 ± 7.42%, n = 158, control, *p* < 0.05; Figure 2B). However, there was no significant difference in the rate of 2-cell embryo formation between the 10 μM treatment group and the control group (85.5 ± 6.38%, n = 170, 10 μM vs. 85.5 ± 7.42%, n = 158, control; Figure 2B). We selected 25 μM BML-277 for further analysis. The rate of 4-cell embryo formation in the early embryos was significantly lower in the 25 μM group than in the control group (16.8 ± 2.22%, n = 236, 25 μM vs. 53.6 ± 7.44%, n = 260, control, *p* < 0.01; Figure 2C). Our results showed that inhibition of CHK2 affected the cleavage of early mouse embryos.

### CHK2 inhibition affected spindle morphology and chromosome alignment at the first cleavage of early mouse embryos

**Figure 3:**
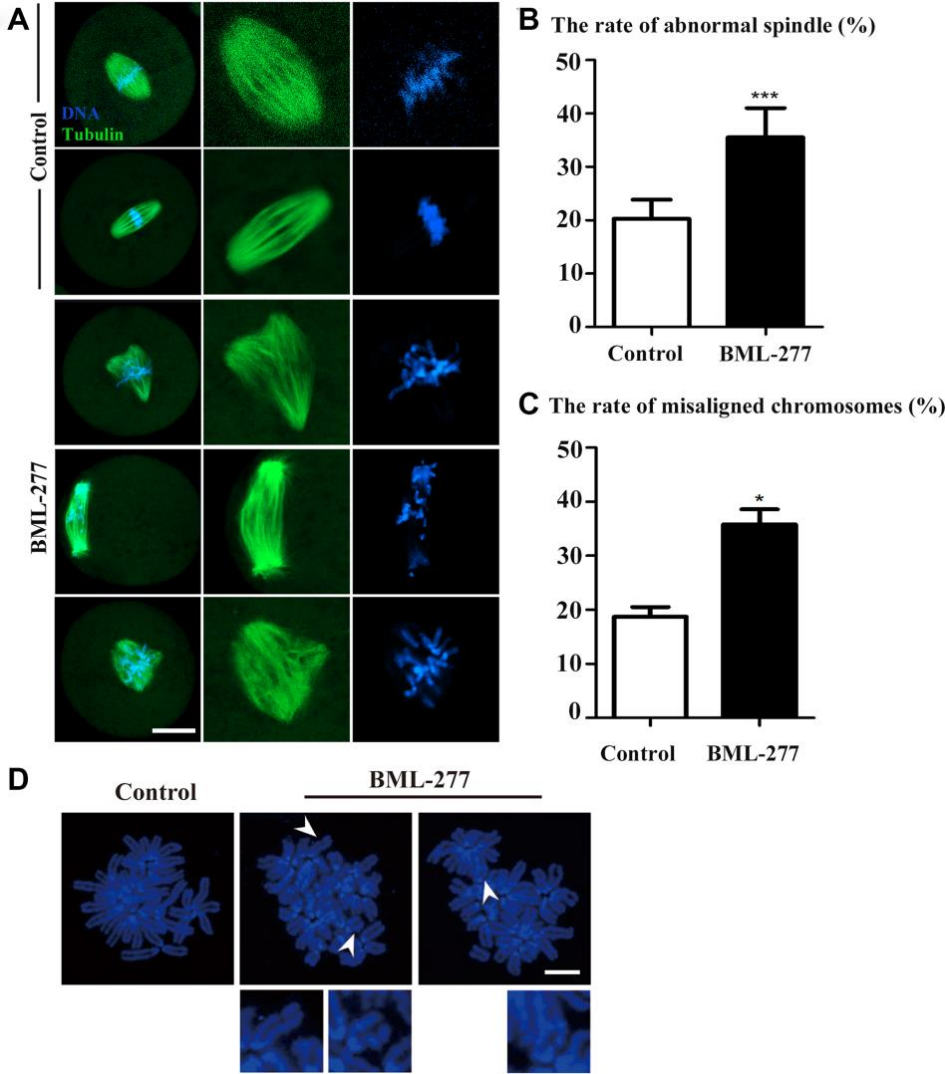
**CHK2 inhibition affected spindle morphology and chromosome alignment at the first cleavage of early mouse embryos.** (**A**) Embryos at metaphase were stained with anti-α-tubulin (green) and counterstained with Hoechst 33342 to visualize chromosomes (blue). Unlike those in the control group, embryos in the 25 μM treatment group showed a variety of defects, including multipolar and nonpolar spindles. The chromosomes in embryos in the 25 μM treatment group were severely misaligned. Bar = 20 μm. (**B**) A significantly higher proportion of embryos in the 25 μM treatment group exhibited spindle defects than those in the control group (35.5 ± 5.51%, n = 173 vs. 20.3 ± 3.55%, n = 165, *p* < 0.001). (**C**) The incidence of chromosome misalignment was also higher in embryos in the 25 μM treatment group than in those in the control group (35.8 ± 2.80%, n = 81 vs. 18.7 ± 1.82%, n = 55, *p* < 0.05). ***significant difference (p < 0.001). *significant difference (*p* < 0.05). (**D**) Chromosomal aberrations were observed in embryos in the CHK2 inhibition group. Blue, DNA. Bar = 5 μm.

Next, we aimed to explore how CHK2 inhibition affects the cleavage of early mouse embryos. Due to the localization pattern of CHK2, we collected embryos at metaphase of the first cleavage to examine spindle morphology. Most embryos in the control group exhibited complete barrel-shaped spindles and well-aligned chromosomes (Figure 3A). In contrast, the spindles of embryos in the 25 μM treatment group showed a variety of defects, including multipolar and nonpolar spindles. In addition, the chromosomes in most embryos in the 25 μM treatment group were severely misaligned (Figure 3A). The incidence of spindle defects in the embryos was significantly higher in the 25 μM treatment group than in the control group (35.5 ± 5.51%, n = 173, 25 μM vs. 20.3 ± 3.55%, n = 165, control, *p* < 0.001; Figure 3B). Similarly, the incidence of chromosome misalignment in the embryos was also higher in the 25 μM treatment group than in the control group (35.8 ± 2.80%, n = 81, 25 μM vs. 18.7± 1.82%, n = 55, control, *p* < 0.05; Figure 3C). We also analyzed chromosome morphology, and the chromosomes in embryos in the 25 μM treatment group showed an aberrant morphology (Figure 3D). These results showed that CHK2 might regulate spindle morphology during development of the early mouse embryo.

### CHK2 inhibition induced DNA damage and oxidative stress in early embryonic development in mice

**Figure 4:**
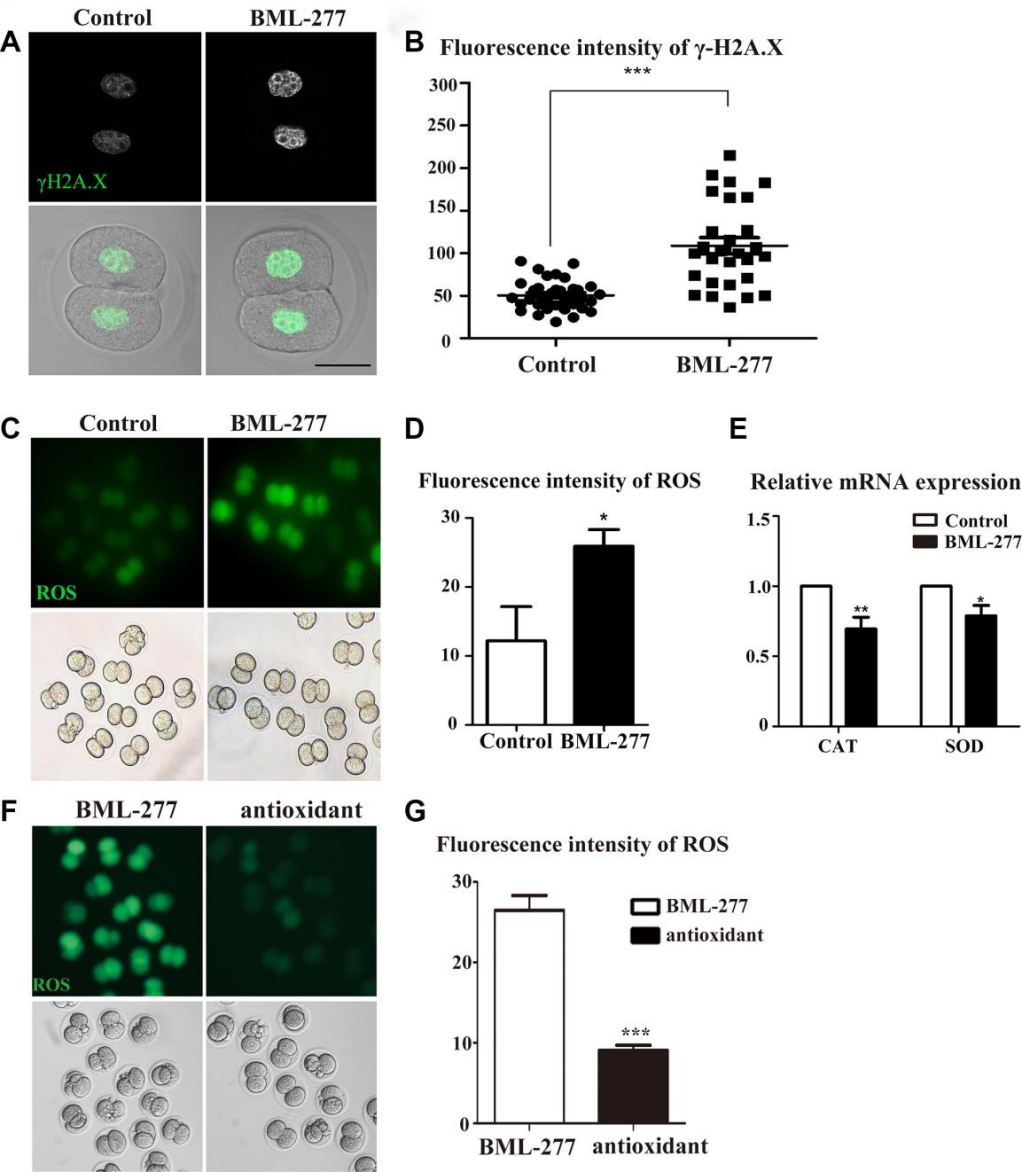
**CHK2 inhibition affected DNA damage and oxidative stress during early embryonic development in mice.** (**A**) Embryos at the 2-cell stage were stained with anti-γ-H2A.X (green). The γ-H2A.X signal was stronger in embryos in the CHK2 inhibition group. Bar = 30 μm. (**B**) The fluorescence intensity of γ-H2A.X in embryos in the 25 μM and control groups (108.8 ± 9.46, n = 28, 25μM vs. 50.7 ± 3.05, n = 32, control, *p* < 0.0001). (**C**) Embryos at the 2-cell stage were stained for ROS (green). The ROS signal was stronger in embryos in the CHK2 inhibition group. (**D**) The fluorescence intensity of ROS in embryos in the 25 μM treatment group and control group (25.9 ± 2.41, n = 43, 25 μM vs. 12.2 ± 5.0, n = 33, control, *p* < 0.05). (**E**) The expression of ROS-related genes in the 25 μM group and control group. (**F**) The ROS signal of embryos in the CHK2 inhibition group decreased after supplementation with an antioxidant. (**G**) ROS fluorescence intensity analysis. ***significant difference (p < 0.001). **significant difference (p < 0.01). *significant difference (p < 0.05).

To further explore the potential regulatory mechanism of CHK2 in early mouse embryos, we used γ-H2A.X as a marker protein to detect the effect of CHK2 on DNA damage during interphase. Immunofluorescent staining results showed that the γ-H2A.X protein was more highly enriched in chromatin in the embryos in the 25 μM treatment group compared with those in the control group (Figure 4A). The fluorescence intensity of γ-H2A.X also confirmed this finding (108.8 ± 9.46, n = 28, 25 μM vs. 50.7 ± 3.05, n = 32, control, *p* < 0.0001; Figure 4B). Since DNA damage can increase intracellular ROS levels, we next explored whether disruption of CHK2 activity would induce oxidative stress in early mouse embryos. We collected embryos at the 2-cell stage for ROS detection in living embryos. The results showed an increase in ROS levels in early embryos after CHK2 activity had been disrupted (Figure 4C). The fluorescence intensity of ROS was also higher in embryos in the 25 μM treatment group than in those in the control group (25.9 ± 2.41, n = 43, 25 μM vs. 12.2 ± 5.0, n = 33, control, *p* < 0.05; Figure 4D). Moreover, we analyzed the expression of genes associated with oxidative stress by RT-PCR. Compared with the control group, the 25 μM group exhibited significantly decreased expression levels of Catalase (CAT) (1.00 vs 0.696 ± 0.083, respectively, *p* < 0.001; Figure 4E) and Superoxide dismutase (SOD) (1.00 vs 0.789 ± 0.074, respectively, *p* < 0.05; Figure 4E). This change in the mRNA expression of genes related to oxidative stress further indicated the occurrence of oxidative stress in the embryos. To further confirm the effects of CHK2 on ROS levels, we also added the antioxidant tempol at a 5 μM concentration to the CHK2 inhibition group. The results showed that the ROS level was reduced compared with that in embryos in the CHK2 inhibition group (Figure 4F), which was confirmed by fluorescence intensity analysis (26.46 ± 1.86 vs 9.06 ± 0.66 in untreated and treated embryos in the CHK2 inhibition group, respectively, *p* < 0.05, Figure 4G). These results suggested that inhibition of CHK2 led to an increase in DNA damage and the occurrence of oxidative stress during early embryonic development in mice.

### CHK2 inhibition induced apoptosis in early mouse embryonic development

**Figure 5:**
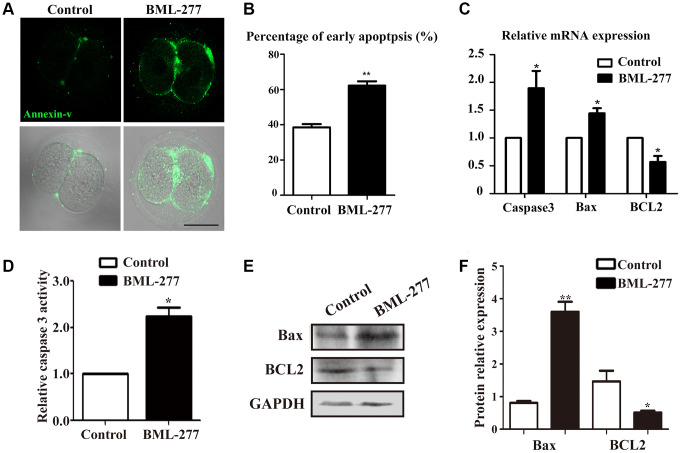
**Inhibition of CHK2 induced apoptosis during early embryonic development in mice.** (**A**) Embryos at the 2-cell stage were stained with Annexin-V (green). The Annexin-V signal was stronger in embryos in the CHK2 inhibition group. Bar = 30 μm. (**B**) The percentages of cells in early apoptosis in mouse embryos treated with BML-277 and mouse embryos in the control group (62.2 ± 2.52%, n = 64, 25 μM vs. 38.6 ± 1.77%, n =92, control, *p* < 0.001). (**C**) The expression of apoptosis-related genes in the 25 μM treatment group and control group. (**D**) Relative Caspase 3 activity in embryos in the control and CHK2 inhibition groups. (**E**) The protein expression of Bax and BCL2 in embryos in the control and CHK2 inhibition groups was determined by immunoblotting. (**F**) Band intensity analysis of Bax and BCL2 in the two groups. **significant difference (p < 0.01). *significant difference (p < 0.05).

Since DNA damage can induce apoptosis, we next performed Annexin-V staining. Positive signals were detected on the cell membrane in embryos in the CHK2 inhibition group, while the few signals observed in the control group indicated the occurrence of early apoptosis (Figure 5A). In the treatment group, the percentage of apoptosis-positive embryos was significantly higher than that in the control group (62.2 ± 2.52%, n = 64, 25 μM vs. 38.6 ± 1.77%, n =92, control, *p* < 0.001; Figure 5B). Moreover, apoptosis-related gene expression was detected by RT-PCR. The expression levels of Compared with the control group, the 25 μM treatment group exhibited significantly increased levels of Caspase 3 (1.00 vs 1.897 ± 0.309, *p* < 0.05; Figure 5C) and Bax (1.00 vs 1.45 ± 0.092, *p* < 0.05; Figure 5C), genes that promote apoptosis. Compared to that in the control group, the expression level of the anti-apoptotic gene BCL2 was much lower in the 25 μM treatment group (1.00 vs 0.569 ± 0.108, *p* < 0.05; Figure 5C). To further confirm the effects of CHK2 on apoptosis control, we also assessed Caspase 3 activity, and the results showed that CHK2 inhibition increased Caspase 3 activity compared with that in the control group (2.25 ± 0.39 vs 1, *p* < 0.05; Figure 5D). Moreover, we also examined Bax and BCL2 protein expression, and Bax expression was increased while BCL2 expression was decreased in the CHK2 inhibition group (Figure 5E). Band intensity analysis also confirmed this finding (Bax: 0.81 ± 0.06 vs 3.6 ± 0.31, *p* < 0.05; BCL2: 1.46 ± 0.33 vs 0.51 ± 0.06, *p* < 0.05; Figure 5F). These results suggested that inhibition of CHK2 induced apoptosis in early mouse embryos.

### CHK2 inhibition induced autophagy in early mouse embryonic development

**Figure 6:**
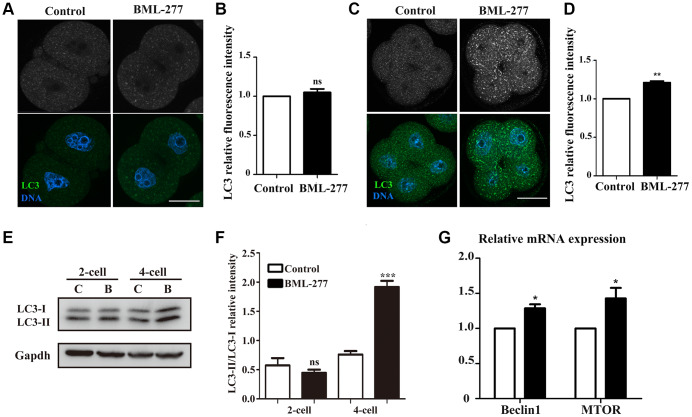
**Inhibition of CHK2 induced autophagy during early embryonic development in mice.** (**A**) Embryos at the 2-cell stage were immunolabeled with anti-LC3 antibody (green), and Hoechst 33342 was used to label DNA (blue). Bar = 30 μm. (**B**) The fluorescence intensity of LC3 in embryos in the 25 μM treatment group and control group. (**C**) Embryos at the 4-cell stage in the control and CHK2 inhibition groups were immunolabeled with anti-LC3 antibody (green), and Hoechst 33342 was used to label DNA (blue). Bar = 30 μm. (**D**) The fluorescence intensity of LC3 in embryos in the 25 μM treatment group and control group. (**E**) The protein expression of LC3-I/II in embryos at the 2-cell and 4-cell stages in the control and CHK2 inhibition groups. (**F**) Band intensity analysis of LC3-I/II in embryos in the control and CHK2 inhibition groups. (**G**) The expression of autophagy-related genes in the 25 μM and control groups. ***significant difference (p < 0.001). **significant difference (p < 0.01). *significant difference (p < 0.05).

Abnormal levels of oxidative stress often lead to apoptosis and further induce autophagy. Next, we collected mouse embryos at the 2-cell stage and 4-cell stage and stained them with LC3 to examine the occurrence of autophagy. We found no significant difference in the LC3 signal in the cytoplasm of 2-cell embryos between the control and treatment groups, which was confirmed by fluorescence intensity analysis (1.00 vs 1.05 ± 0.44; Figure 6A and 6B). However, 4-cell embryos in the CHK2 inhibition group showed more autophagosomes than 4-cell embryos in the control group (Figure 6C). The relative fluorescence intensity of LC3 was also higher in embryos in the 25 μM treatment group than in the control embryos (1 vs 1.21 ± 0.02, *p* < 0.001; Figure 6D). We also examined the LC3 protein expression level, and the results were consistent with those of fluorescence staining (Figure 6E), which was confirmed by LC3-II/I band intensity analysis (0.57 ± 0.13, lane 1 vs. 0.45 ± 0.05, lane 2, *p* > 0.05; 0.76 ± 0.06, lane 3 vs. 1.92 ± 0.11, lane 4, *p* < 0.001) (Figure 6F). Moreover, expression levels of the autophagy-related genes MTOR (1 vs 1.43 ± 0.15, *p* < 0.05; Figure 5H) and Beclin 1 (1 vs 1.29 ± 0.06, *p* < 0.05; Figure 5H) were higher in the 25 μM treatment group than in the control group. These results showed that CHK2 inhibition induced autophagy during early embryonic development in mice.

## DISCUSSION

In this study, we investigated the potential functions of CHK2 during early embryonic development in mice. We found that CHK2 regulates spindle formation during the first cleavage of embryonic development. Moreover, disruption of CHK2 activity caused DNA damage and further induced intracellular oxidative stress, which caused apoptosis and autophagy. Our results provide evidence that CHK2 is a critical regulator of early embryonic development in mice.

In mouse oocytes, CHK2 was shown to localize at the spindle poles [24], while our results showed a similar CHK2 localization pattern in mouse embryos and that the loss of CHK2 activity caused defects in early embryonic development in mice. Previous studies have also shown that CHK2 is involved in regulating cell cycle progression in both meiosis and mitosis [24, 25]. The pattern of CHK2 localization in mouse embryos indicates its roles in spindle dynamics. Our results showed that CHK2 inhibition led to the disruption of spindle organization and the abnormal arrangement of chromosomes at metaphase in the first cleavage of the early mouse embryo. Assembly of the spindle is mainly dependent on microtubules [26], which are involved in the regulation of cell shape, cell movement, cell transport and cell division [27]. Abnormal arrangement and segregation of chromosomes may cause aneuploidy in embryos, further leading to implantation failure, spontaneous abortion or embryo death [28]. In colorectal cancer cells, the absence of CHK2 affected mitotic microtubule assembly, which led to chromosomal instability [29, 30]. CHK2 inhibition also resulted in spindle defects and chromosome misalignment in mouse oocytes [24]. Our results are consistent with these previous reports, revealing the conserved functions of CHK2 in monitoring spindle assembly and chromosome alignment in different models and species.

Endogenous DNA damage induced by replication errors and DNA demethylation has been proven to actively occur in zygotes [31, 32]. DNA damage can affect the integrity of the biological genome [33]. The DNA damage response (DDR) system repairs DNA damage or eliminate cells that cannot be repaired [34] and involves cell cycle arrest, DNA repair or the initiation of apoptosis [35]. As a checkpoint kinase, CHK2 is involved in DNA repair, cell cycle arrest and apoptosis in cells [36]. CHK2 dysfunction results in checkpoint supervision failure and DNA damage in cells, usually accompanied by mitotic defects [37–39]. A previous study also showed that Cds1 (a CHK2 homologue) is activated by DNA damage and phosphorylates CDC2 to regulate the S/M or G2/M transition in *S. pombe* [40]. CHK2 was also shown to have necessary functions in repairing DNA damage by its phosphopeptide-binding ability in the early *Drosophila* embryo [41]. Our results are similar to those of earlier reports and show that inhibition of CHK2 resulted in an increase in γ-H2A.X, indicating the occurrence of DNA damage, and that CHK2 is involved in DNA repair in early mouse embryonic development.

In addition, DNA damage can increase the level of reactive oxygen species (ROS) [42, 43]. Specifically, the histone H2A variant H2AX accumulates in cells with DNA damage. Overexpression of H2AX increases the activity of NADP(H) oxidase (Nox), which in turn elevates the level of ROS [42]. Our results showed that the loss of CHK2 activity induced ROS generation, and changes in the expression of ROS-related genes further confirmed the occurrence of oxidative stress. This defect could be rescued by supplementation with the antioxidant tempol. Therefore, a high level of oxidative stress might contribute to the early embryonic defects following CHK2 inhibition observed in the mice. Irreparable DNA damage and oxidative stress usually activate apoptosis and autophagy to clear damaged cells. CHK2 was shown to be involved in regulating the p53-mediated apoptotic response in mouse embryo fibroblasts [23]. Our results also showed that the inhibition of CHK2 enhanced the apoptotic signal and increased the number of intracellular autophagic vesicles in early mouse embryos, indicating the occurrence of apoptosis and autophagy. Therefore, CHK2 might monitor DNA damage to avoid oxidative stress, preventing apoptosis and autophagy in mouse embryos.

In conclusion, our results indicated that CHK2 is essential for early mouse embryonic development through its regulation of spindle assembly, chromosome alignment and DNA repair.

## MATERIALS AND METHODS

### Antibodies and chemicals

The CHK2 inhibitor BML-277 was purchased from Merck and Millipore (USA). Rabbit polyclonal anti-CHK2 antibody, rabbit monoclonal anti-γ-H2A.X antibody and anti-MAP1LC3A antibody were purchased from Abcam (Cambridge, UK). Anti-α-tubulin-FITC antibody and Hoechst 33342 were purchased from Sigma (St. Louis, MO, USA). Alexa Fluor 488 goat anti-rabbit antibody and Alexa Fluor 594 goat anti-rabbit antibody were purchased from Invitrogen (Carlsbad, CA, USA).

### Parthenogenetic activation of oocytes and embryo culture

All animal experiments followed the standards set by the Animal Care and Use Committee of Nanjing Agriculture University. Female ICR mice aged 6-8 weeks were intraperitoneally injected with 5 IU pregnant mare serum gonadotropin (PMSG) (Ningbo Second Hormone Factory, China). At 48 h after PMSG injection, the mice were injected with 5 IU human chorionic gonadotropin (hCG) (Ningbo Second Hormone Factory). Cumulus oocyte complexes (COCs) were collected from the ampullae of the oviducts at 16 h after the injection of hCG and then treated with 0.1% hyaluronidase at 37°C for 5 min. Then, the denuded MII oocytes were placed into chemical parthenogenetic activation medium and cultured for 5 h. Cytochalasin B (CB, Abcam, 5 μg/ml), 2 mM ethylene glycol-bis(2-aminoethyl ether)-N,N,N’,N’-tetraacetic acid (EGTA, Solarbio), and 5 mM SrCl_2_ (Sigma) were added to M16 medium (Sigma) to form chemical parthenogenetic activation medium. Oocytes that showed the presence of male and female pronuclei were selected as zygotes. The zygotes were transplanted into fresh M16 culture medium under mineral oil and cultured at 37°C in a 5% CO_2_ atmosphere.

### BML-277 treatment

BML-277, an ATP-competitive inhibitor of CHK2, can effectively inhibit the activity of CHK2 *in vivo*. Therefore, we chose BML-277 to study the function of CHK2 in early embryonic development in mice. BML-277 was dissolved in DMSO to a concentration of 10 mM as a storage solution and then diluted to working concentrations of 10 μM and 25 μM in M16 medium. These concentrations were chosen based on previous studies. Control embryos were cultured in fresh M16 medium. We cultured the embryos for 24 h to obtain embryos at the 2-cell stage and 48 h to obtain embryos at the 4-cell stage.

### Immunofluorescence staining and confocal microscopy

For single staining of CHK2, tubulin, γ-H2A.X or LC3A, embryos were fixed in 4% paraformaldehyde (PFA) for 30 min and then permeabilized with 0.5% Triton X-100 for 20 min at room temperature, followed by blocking in 1% BSA-supplemented PBS at room temperature for 1 h. Subsequently, the embryos were incubated with different primary antibodies (anti-CHK2, 1:20; anti-α-tubulin-FITC, 1:100; anti-γ-H2A.X, 1:200; anti-MAP1LC3A, 1:100) for 24 h at 4°C. The embryos were further incubated with the corresponding secondary antibodies (Alexa Fluor 488-conjugated goat anti-rabbit or Alexa Fluor 594-conjugated goat anti-rabbit antibody, 1:200) for 1 h at room temperature. Finally, all embryos were stained with Hoechst 33342 (10 mg/mL in PBS) for 10 min at room temperature, and the samples were mounted on glass slides and detected with a laser-scanning confocal fluorescence microscope (Zeiss LSM 800 META, Germany).

### Reactive oxygen species (ROS) level detection

A Reactive Oxygen Species Assay Kit (DCFH-DA, Beyotime Institute of Biotechnology, China) was used to analyze the ROS levels in viable embryos at the 2-cell stage. The viable embryos were incubated with DCFH-DA (1:800) in fresh M16 medium for 30 min at 37°C, transferred into preheated fresh M16 medium and washed three times. A fluorescence microscope (Olympus CKX53, Japan) was adopted to detect ROS fluorescent signals, and the fluorescence intensities were analyzed by ImageJ software (NIH).

### Annexin-V staining

For Annexin-V staining, Annexin-V-FITC (1:10, Vazyme Biotech Co., Ltd., Nanjing, China) and Hoechst 33342 (1:500) were diluted with M16 medium. Then, living embryos were placed in the medium for 30 min at 37°C. Subsequently, the embryos were fixed in 4% paraformaldehyde for 30 min, permeabilized with 0.5% Triton X-100 for 20 min and blocked in 1% BSA-supplemented PBS at room temperature for 1 h. Finally, the samples were mounted on glass slides and detected with a laser-scanning confocal fluorescence microscope (Zeiss LSM 800 META, Germany).

### Caspase 3 activity assay

Caspase 3 activity in early embryos treated with BML-277 was qualified with a Caspase 3/CPP32 colorimetric assay kit (BioVision, CA, USA) according to the manufacturer’s instructions. Briefly, we transferred embryos to a chilled cell lysis buffer for protein production and measured the concentration. Then, we added an equal volume of 2× reaction buffer (containing 10 mM DTT) and DEVD-pNA substrate and incubated the embryos for 1 h at 37°C. The absorbance at 405 nm was measured in a microplate reader (Thermo LabSystems).

### Real-time quantitative PCR analysis

Real-time quantitative PCR was used to analyze the mRNA expression of ROS-, apoptosis- and autophagy-related genes. Total RNA was extracted from 30 embryos at the 2-cell stage using a Dynabeads mRNA DIRECT kit (Invitrogen Dynal AS, Norway). PrimeScript RT Master Mix (Takara, Japan) was used to generate first-strand cDNA. Each 20 μl RT-PCR sample consisted of 10 μl of Fast Universal SYBR Green Master (ROX), 0.8 μl of both forward primer and reverse primer, 1 μl of cDNA, and 7.4 μl of ddH_2_O. Real-time quantitative PCR was conducted with a fast-real-time PCR system (ABI Step One Plus). GAPDH was used as a reference gene, and relative gene expression levels were analyzed by the 2^−ΔΔCt^ method. The sequences of primers for related genes are listed in Table 1.

**Table 1:** Primer sequences for RT-PCR.

**Gene**	**Forward primer**	**Reverse primer**
CAT	5'-GCAGATACCTGTGAACTGTC-3'	5'-GTAGAATGTCCGCACCTGAG-3'
SOD	5’-AAAGCGGTGTGCGTGCTGAA-3’	5’-CAGGTCTCCAACATGCCTCT-3’
BAX	5’-TGAAGACAGGGGCCTTTTTG-3’	5’-AATTCGCCGGAGACACTCG-3'
BCL2	5’-ATGCCTTTGTGGAACTATATGGC-3’	5’-GGTATGCACCCAGAGTGATGC-3'
Caspase3	5’-ATGGAGAACAACAAAACCTCAGT-3’	5’-TTGCTCCCATGTATGGTCTTTAC-3'
Beclin-1	5’-ATGGAGGGGTCTAAGGCGTC-3'	5’-TCCTCTCCTGAGTTAGCCTCT-3
mTOR	5’-ACCGGCACACATTTGAAGAAG-3'	5’-CTCGTTGAGGATCAGCAAGG-3'
GAPDH	5’-AGGTCGGTGTGAACGGATTTG-3'	5’-TGTAGACCATGTAGTTGAGGTCA-3'

### Statistical analysis

All experimental data were obtained from at least three repeated experiments. Means ± standard errors (SEMs) are used to express the results of the groups. GraphPad Prism 5 software was used for statistical analyses, and *P* < 0.05 indicated statistical significance.

## References

[1] Duan X, Chen KL, Zhang Y, Cui XS, Kim NH, Sun SC. ROCK inhibition prevents early mouse embryo development. Histochem Cell Biol. 2014; 142:227–33. 10.1007/s00418-014-1201-6 24562870

[2] Yurttas P, Morency E, Coonrod SA. Use of proteomics to identify highly abundant maternal factors that drive the egg-to-embryo transition. Reproduction. 2010; 139:809–23. 10.1530/REP-09-0538 20106898

[3] Marikawa Y, Alarcón VB. Establishment of trophectoderm and inner cell mass lineages in the mouse embryo. Mol Reprod Dev. 2009; 76:1019–32. 10.1002/mrd.21057 19479991 PMC2874917

[4] Schafer KA. The cell cycle: a review. Vet Pathol. 1998; 35:461–78. 10.1177/030098589803500601 9823588

[5] Barnum KJ, O’Connell MJ. Cell cycle regulation by checkpoints. Methods Mol Biol. 2014; 1170:29–40. 10.1007/978-1-4939-0888-2_2 24906307 PMC4990352

[6] O’Connell MJ, Walworth NC, Carr AM. The G2-phase DNA-damage checkpoint. Trends Cell Biol. 2000; 10:296–303. 10.1016/s0962-8924(00)01773-6 10856933

[7] Giono LE, Manfredi JJ. The p53 tumor suppressor participates in multiple cell cycle checkpoints. J Cell Physiol. 2006; 209:13–20. 10.1002/jcp.20689 16741928

[8] Latif C, den Elzen NR, O’Connell MJ. DNA damage checkpoint maintenance through sustained Chk1 activity. J Cell Sci. 2004; 117:3489–98. 10.1242/jcs.01204 15213253

[9] Tapia-Alveal C, Calonge TM, O’Connell MJ. Regulation of chk1. Cell Div. 2009; 4:8. 10.1186/1747-1028-4-8 19400965 PMC2685127

[10] Musacchio A, Salmon ED. The spindle-assembly checkpoint in space and time. Nat Rev Mol Cell Biol. 2007; 8:379–93. 10.1038/nrm2163 17426725

[11] Sun SC, Kim NH. Spindle assembly checkpoint and its regulators in meiosis. Hum Reprod Update. 2012; 18:60–72. 10.1093/humupd/dmr044 22086113

[12] Jeganathan KB, van Deursen JM. Differential mitotic checkpoint protein requirements in somatic and germ cells. Biochem Soc Trans. 2006; 34:583–86. 10.1042/BST0340583 16856867

[13] Wei L, Liang XW, Zhang QH, Li M, Yuan J, Li S, Sun SC, Ouyang YC, Schatten H, Sun QY. BubR1 is a spindle assembly checkpoint protein regulating meiotic cell cycle progression of mouse oocyte. Cell Cycle. 2010; 9:1112–21. 10.4161/cc.9.6.10957 20237433

[14] Tunquist BJ, Eyers PA, Chen LG, Lewellyn AL, Maller JL. Spindle checkpoint proteins Mad1 and Mad2 are required for cytostatic factor-mediated metaphase arrest. J Cell Biol. 2003; 163:1231–42. 10.1083/jcb.200306153 14691134 PMC2173727

[15] Matsuoka S, Huang M, Elledge SJ. Linkage of ATM to cell cycle regulation by the Chk2 protein kinase. Science. 1998; 282:1893–97. 10.1126/science.282.5395.1893 9836640

[16] Kim ST, Lim DS, Canman CE, Kastan MB. Substrate specificities and identification of putative substrates of ATM kinase family members. J Biol Chem. 1999; 274:37538–43. 10.1074/jbc.274.53.37538 10608806

[17] Durocher D, Jackson SP. The FHA domain. FEBS Lett. 2002; 513:58–66. 10.1016/s0014-5793(01)03294-x 11911881

[18] Ahn J, Urist M, Prives C. The Chk2 protein kinase. DNA Repair (Amst). 2004; 3:1039–47. 10.1016/j.dnarep.2004.03.033 15279791

[19] Antoni L, Sodha N, Collins I, Garrett MD. CHK2 Kinase: cancer susceptibility and cancer therapy - two sides of the same coin? Nat Rev Cancer. 2007; 7:925–36. 10.1038/nrc2251 18004398

[20] Zhou BB, Elledge SJ. The DNA damage response: putting checkpoints in perspective. Nature. 2000; 408:433–39. 10.1038/35044005 11100718

[21] Xu J, Du W. Drosophila chk2 plays an important role in a mitotic checkpoint in syncytial embryos. FEBS Lett. 2003; 545:209–12. 10.1016/s0014-5793(03)00536-2 12804777

[22] Stolz A, Ertych N, Bastians H. Tumor suppressor CHK2: regulator of DNA damage response and mediator of chromosomal stability. Clin Cancer Res. 2011; 17:401–05. 10.1158/1078-0432.CCR-10-1215 21088254

[23] Jack MT, Woo RA, Hirao A, Cheung A, Mak TW, Lee PW. Chk2 is dispensable for p53-mediated G1 arrest but is required for a latent p53-mediated apoptotic response. Proc Natl Acad Sci USA. 2002; 99:9825–29. 10.1073/pnas.152053599 12097646 PMC125030

[24] Dai XX, Duan X, Liu HL, Cui XS, Kim NH, Sun SC. Chk2 regulates cell cycle progression during mouse oocyte maturation and early embryo development. Mol Cells. 2014; 37:126–32. 10.14348/molcells.2014.2259 24598997 PMC3935625

[25] Zeng Y, Forbes KC, Wu Z, Moreno S, Piwnica-Worms H, Enoch T. Replication checkpoint requires phosphorylation of the phosphatase Cdc25 by Cds1 or Chk1. Nature. 1998; 395:507–10. 10.1038/26766 9774107

[26] Tang F, Pan MH, Lu Y, Wan X, Zhang Y, Sun SC. Involvement of Kif4a in spindle formation and chromosome segregation in mouse oocytes. Aging Dis. 2018; 9:623–33. 10.14336/AD.2017.0901 30090651 PMC6065292

[27] Nogales E. Structural insights into microtubule function. Annu Rev Biochem. 2000; 69:277–302. 10.1146/annurev.biochem.69.1.277 10966460

[28] Wei Y, Multi S, Yang CR, Ma J, Zhang QH, Wang ZB, Li M, Wei L, Ge ZJ, Zhang CH, Ouyang YC, Hou Y, Schatten H, Sun QY. Spindle assembly checkpoint regulates mitotic cell cycle progression during preimplantation embryo development. PLoS One. 2011; 6:e21557. 10.1371/journal.pone.0021557 21720555 PMC3123354

[29] Ertych N, Stolz A, Stenzinger A, Weichert W, Kaulfuß S, Burfeind P, Aigner A, Wordeman L, Bastians H. Increased microtubule assembly rates influence chromosomal instability in colorectal cancer cells. Nat Cell Biol. 2014; 16:779–91. 10.1038/ncb2994 24976383 PMC4389786

[30] Ertych N, Stolz A, Valerius O, Braus GH, Bastians H. CHK2-BRCA1 tumor-suppressor axis restrains oncogenic aurora-A kinase to ensure proper mitotic microtubule assembly. Proc Natl Acad Sci USA. 2016; 113:1817–22. 10.1073/pnas.1525129113 26831064 PMC4763769

[31] Ménézo Y, Dale B, Cohen M. DNA damage and repair in human oocytes and embryos: a review. Zygote. 2010; 18:357–65. 10.1017/S0967199410000286 20663262

[32] Wossidlo M, Arand J, Sebastiano V, Lepikhov K, Boiani M, Reinhardt R, Schöler H, Walter J. Dynamic link of DNA demethylation, DNA strand breaks and repair in mouse zygotes. EMBO J. 2010; 29:1877–88. 10.1038/emboj.2010.80 20442707 PMC2885932

[33] Palou R, Palou G, Quintana DG. A role for the spindle assembly checkpoint in the DNA damage response. Curr Genet. 2017; 63:275–80. 10.1007/s00294-016-0634-y 27488803 PMC5383677

[34] Ciccia A, Elledge SJ. The DNA damage response: making it safe to play with knives. Mol Cell. 2010; 40:179–204. 10.1016/j.molcel.2010.09.019 20965415 PMC2988877

[35] Zannini L, Delia D, Buscemi G. CHK2 kinase in the DNA damage response and beyond. J Mol Cell Biol. 2014; 6:442–57. 10.1093/jmcb/mju045 25404613 PMC4296918

[36] Hirao A, Cheung A, Duncan G, Girard PM, Elia AJ, Wakeham A, Okada H, Sarkissian T, Wong JA, Sakai T, De Stanchina E, Bristow RG, Suda T, et al. Chk2 is a tumor suppressor that regulates apoptosis in both an ataxia telangiectasia mutated (ATM)-dependent and an ATM-independent manner. Mol Cell Biol. 2002; 22:6521–32. 10.1128/mcb.22.18.6521-6532.2002 12192050 PMC135625

[37] Reinhardt HC, Yaffe MB. Kinases that control the cell cycle in response to DNA damage: Chk1, Chk2, and MK2. Curr Opin Cell Biol. 2009; 21:245–55. 10.1016/j.ceb.2009.01.018 19230643 PMC2699687

[38] Castedo M, Perfettini JL, Roumier T, Yakushijin K, Horne D, Medema R, Kroemer G. The cell cycle checkpoint kinase Chk2 is a negative regulator of mitotic catastrophe. Oncogene. 2004; 23:4353–61. 10.1038/sj.onc.1207573 15048074

[39] Bartek J, Falck J, Lukas J. CHK2 kinase—a busy messenger. Nat Rev Mol Cell Biol. 2001; 2:877–86. 10.1038/35103059 11733767

[40] McGowan CH. Checking in on Cds1 (Chk2): a checkpoint kinase and tumor suppressor. Bioessays. 2002; 24:502–11. 10.1002/bies.10101 12111733

[41] Takada S, Collins ER, Kurahashi K. The FHA domain determines drosophila Chk2/mnk localization to key mitotic structures and is essential for early embryonic DNA damage responses. Mol Biol Cell. 2015; 26:1811–28. 10.1091/mbc.E14-07-1238 25808488 PMC4436828

[42] Kang MA, So EY, Simons AL, Spitz DR, Ouchi T. DNA damage induces reactive oxygen species generation through the H2AX-Nox1/Rac1 pathway. Cell Death Dis. 2012; 3:e249. 10.1038/cddis.2011.134 22237206 PMC3270268

[43] Rowe LA, Degtyareva N, Doetsch PW. DNA damage-induced reactive oxygen species (ROS) stress response in saccharomyces cerevisiae. Free Radic Biol Med. 2008; 45:1167–77. 10.1016/j.freeradbiomed.2008.07.018 18708137 PMC2643028

